# Surgical treatment of congenital diaphragmatic hernia in a single institution

**DOI:** 10.1186/s13019-022-02098-w

**Published:** 2022-12-30

**Authors:** Hua Li, Shengliang Zhao, Chun Wu, Zhengxia Pan, Gang Wang, Jiangtao Dai

**Affiliations:** 1 Children’s Hospital of Chongqing Medical University; Department of Cardio-Thoracic Surgery; Ministry of Education Key Laboratory of Child Development and Disorders; National Clinical Research Center for Child Health and Disorders; China International Science and Technology Cooperation base of Child development and Critical Disorders;Chongqing Key laboratory of Pediatrics, Chongqing, 400037 People’s Republic of China; 2 Department of Thoracic Surgery, Xinqiao Hospital, Army Medical University (Third Military Medical University), Chongqing, 400037 People’s Republic of China; 3 Department of Cardio-Thoracic Surgery, Children’s Hospital of Chongqing Medical University, No. 20, Jinyu Avenue, Liangjiang New District, Chongqing, People’s Republic of China

**Keywords:** Congenital diaphragmatic hernia, Minimally invasive surgery, Risk stratification, Thoracoscopic, Infants

## Abstract

**Background:**

This study aimed to evaluate the effectiveness of video-assisted thoracic surgery for the treatment of congenital diaphragmatic hernia (CDH) in a larger series compared with conventional open surgery. Additionally, we summarized the experience of thoracoscopic surgery in the treatment of CDH in infants.

**Methods:**

We retrospectively analysed the clinical data of 109 children with CDH who underwent surgical treatment at the Department of Cardiothoracic Surgery of Children’s Hospital of Chongqing Medical University from January 2011 to January 2021. According to the surgical method, the children were divided into an open group (62 cases) and a thoracoscopy group (47 cases).Patients who underwent surgical correction had the diaphragmatic defect size graded (A–D) using a standardized system. We compared the operation time, intraoperative blood loss, postoperative mechanical ventilation time, postoperative hospital stay, postoperative CCU admission time and other surgical indicators as well as the recurrence rate, mortality rate and complication rate of the two groups of children.

**Results:**

The index data on the operation time, intraoperative blood loss, postoperative mechanical ventilation time, postoperative hospital stay and postoperative CCU admission time were better in the thoracoscopy group than in the open group. The difference between the two groups was statistically significant (*P* < 0.05). We compared the number of incision infections, lung infections, atelectasis, pleural effusion, and chylothorax between the two groups. There were more children in the open group than in the thoracoscopy group. The overall incidence of postoperative complications in the open group (51.61%) was higher than that in the thoracoscopy group (44.68%).The recurrence rate of the thoracoscopy group (8.51%) was higher than that of the open group (3.23%). In the open group, 7 patients died of respiratory distress after surgery, and no patients died in the thoracoscopy group.

**Conclusions:**

Thoracoscopic surgery and open surgery can effectively treat CDH. Compared with conventional open surgery, thoracoscopy has the advantages of shorter operation time, less trauma, faster recovery and fewer complications. We believe that thoracoscopic surgery for type A/B diaphragmatic defect has certain advantages, but there is a risk of recurrence.

## Background

Congenital diaphragmatic hernia (CDH) is due to developmental disorders or dysplasia of the diaphragm. It does not become clear that this happens during early embryonic development. The contents of the abdominal cavity herniate into the thoracic cavity and compress the lungs and heart, which can lead to pulmonary dysplasia and pulmonary hypertension. The incidence of CDH is approximately 1:2500–1:5000 [1], and the fatality rate of CDH is as high as 40–60% [2, 3]. The CDH phenotype with its clinical implications is very diverse, so there are different surgical methods. The current treatment still requires surgical repair of the diaphragm defect, including open surgery and minimally invasive surgery. Open surgery generally involves traditional laparotomy or thoracotomy, and minimally invasive surgery (MIS) is divided into laparoscopic and thoracoscopic surgery. Related studies have suggested that the operation time, hospitalization time, postoperative mechanical ventilation time and postoperative analgesic use of children after MIS are better than those after open surgery but that it may cause hypercapnia and hypoxia [4]. Additionally, the recurrence rate after MIS is also higher [5]. However, comparative studies between MIS and open surgery have not been conducted extensively due to wide discrepancies in the severity of pulmonary hypoplasia and pulmonary hypertension between patients as well as different management protocols, resulting in the indications for MIS varying from centre to centre. We selected 109 children with CDH as the research subjects to observe the comparison of thoracoscopic diaphragmatic hernia repair with traditional open surgery to provide a reference basis for clinical decision-making regarding CDH surgery.

## Methods

We retrospectively analysed the clinical data of 109 children with CDH admitted to the Department of Cardiothoracic Surgery, Children's Hospital of Chongqing Medical University from January 2011 to January 2021. The Medical Research Ethics Committee of Children's Hospital Affiliated to Chongqing Medical University approved the study, and this study obtained written informed consent from the families of all children. The study protocol is performed in accordance with the relevant guidelines.

**Fig. 1:**
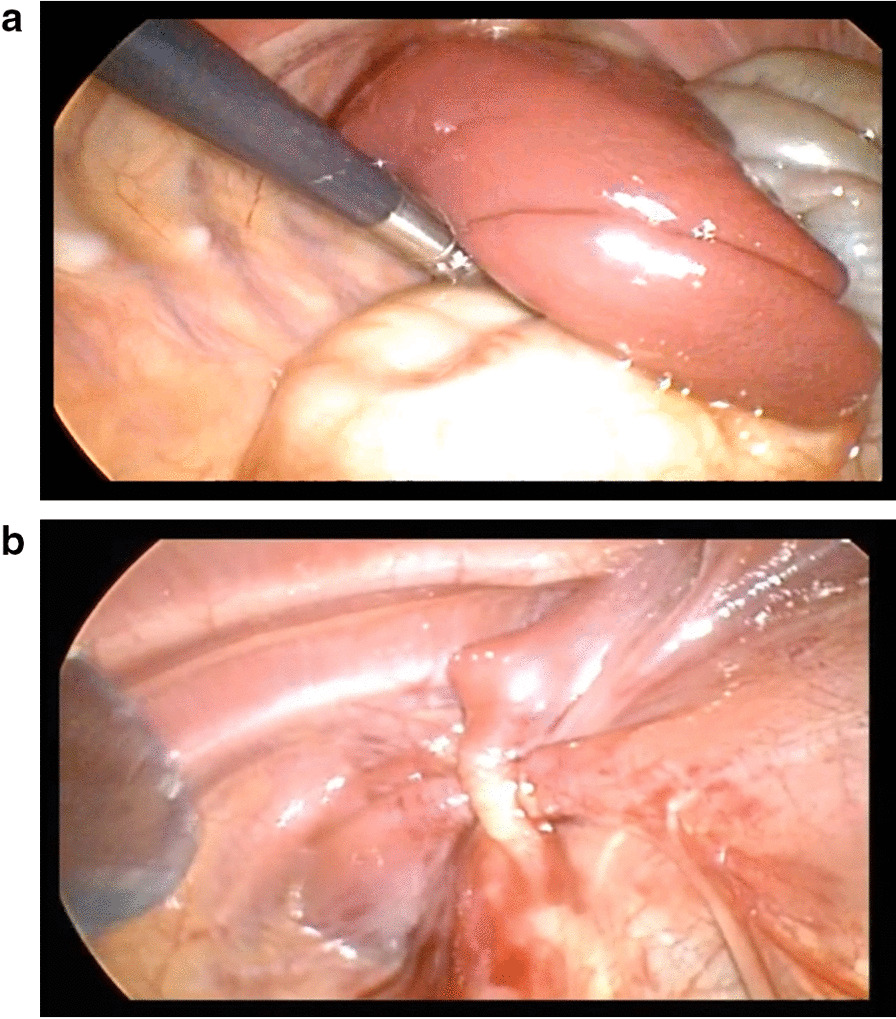
Intraoperative and postoperative images of congenital diaphragmatic hernia repair under thoracoscopy

In the open surgery group, through the thoracoabdominal approach, we removed the weak diaphragm and used intermittent nonabsorbable sutures to ensure that the cut diaphragm had a shingled shape to strengthen the weak area of the diaphragm. We once again sutured the ventral muscle layer of the diaphragm with intermittent mattresses. If there is a hernia sac, we will remove it at the same time. For the thoracoscopic group, a 5 mm trocar was placed on the lower edge of the scapula tip using the three-hole method, and two operation holes were made in the fourth intercostal space on both sides of the trocar. The thoracic cavity pressurizes the abdominal organs into the abdominal cavity and fully exposes the diaphragm defect to observe the size of the diaphragm defect. If there was a hernia sac in the child, it was pushed into the abdominal cavity, and a purse-string suture with a needled suture was used to repair the diaphragmatic defect, close the CDH, restabilize and suture the edge of the defect with barbed sutures. In addition, a chest drainage tube was placed after surgery. See Fig. 1.

Patients who underwent surgical correction had the diaphragmatic defect size graded (A–D) using a standardized system.Defects were coded from A to D. “A” defects were entirely surrounded by muscle, “B” defects had a small (b50%) and “C” defects a large (N50%) portion of the chest wall devoid of diaphragm tissue, and “D” patients had complete or near complete absence of the diaphragm [6]. We observed the operation time, intraoperative blood loss, postoperative mechanical ventilation time, postoperative hospital stay, postoperative CCU admission time and other surgical indicators as well as the recurrence rate, mortality rate and complication rate of the two groups of children.

### Statistical analyses

All the collected data were statistically analyzed using SPSS 22.0 software. The continuous variables were expressed as the mean ± standard deviation, and the classification variables were expressed as ratio columns. The comparison between the two groups was expressed by independent sample t-tests, and the count data were expressed by Fisher's precision test. The difference was statistically significant with a *P* value of < 0.05.

## Results

This study included 109 children with CDH. According to different surgical methods, 47 children who underwent thoracoscopic diaphragmatic hernia repair were included in the thoracoscopy group, and 62 children who underwent traditional open surgery were included in the open group. In the open group, 56 patients underwent transabdominal diaphragmatic hernia repair, and 6 patients underwent transthoracic hernia repair. There were 47 cases in the thoracoscopy group, including 25 males and 22 females who were aged 1 h to 1 year and 2 months (average of 9.05 ± 3.97 months) and weighed 2.48–11.2 kg (average of 6.55 ± 2.44 kg). With regard to the location of the disease, 38 cases on the left side, and 9 cases on the right side. There were 62 patients in the open group, including 36 males and 26 females who were aged 2 h to 2 years (average of 8.83 ± 6.11 months) and weighed 2.51–14.2 kg (average of 7.04 ± 3.73 kg). With regard to the location of the disease, 51 cases were on the left side, and 11 cases were on the right side. In the open group, 10 cases were complicated with other malformations, including 3 cases of cryptorchidism, 2 cases of pectus excavatum and 5 cases of right heart shift. In the thoracoscopic group, 15 cases were complicated with other malformations, including 5 cases of cryptorchidism, 7 cases of pectus excavatum and 3 cases of right heart shift. The types of diaphragmatic defects in the thoracoscopy group and the open group are shown in Table 1, and the difference between the two groups was statistically significant (*P* < 0.05). There was no significant difference in sex, age, weight, location of disease or other preoperative data between the two groups (*P* > 0.05), indicating that the two groups were comparable (Table 1). In the thoracoscopy group, there were 5 children with definite prenatal diagnosis, and 9 children in the open group had definite prenatal diagnosis. The dead children were not included in this study.

**Table 1:** Comparison of general clinical data of the two groups of children (n = 109)

General information	Open group (n = 62)	Thoracoscopy group (n = 47)	*P* value
Gender (male: female)	36:26	25:22	0.612
Age of operation (months)	8.83 ± 6.11	9.05 ± 3.97	0.755
Weight (kg)	7.04 ± 3.73	6.55 ± 2.44	0.470
Location of diaphragmatic hernia (left: right)	51:11	38:9	0.851
Combined malformation (congenital heart disease: congenital pulmonary dysplasia: others)	29:33:10	16:20:15	0.106
Use of nitric oxide	4(6.45%)	1(2.13%)	0.388
Use of HFO	3(4.84%)	1(2.13%)	0.633
ECMO	4(6.45%)	0	0.132
Defect A:Defect B:Defect C:Defect D	17:21:23:1	28:17:2:0	0.000

All children in the open group completed the operation successfully, and 3 cases in the thoracoscopy group were converted to open surgery due to severe thoracic adhesion and abdominal organs obstructing the surgical field. During the operation, the orifice diameter of the CDH was 2–12 cm, and the average area of the diaphragmatic muscle defect was 18.12 ± 6.43 cm^2^. The 89 cases with left CDH mainly herniated into the following organs: small intestine, stomach, colon, mesenteric and spleen. The 20 cases of right CDH mainly herniated into the right lobe of the liver, small intestine and colon.

We analysed the data of the relevant surgical indicators in the two groups. The index data of operation time, intraoperative blood loss, postoperative mechanical ventilation time, postoperative hospital stay, postoperative CCU admission time and other indicators in the thoracoscopy group were better than those in the open group. The difference between the two groups was statistically significant (*P* < 0.05) (Table 2). Compared with the number of incision infections, pulmonary infections, atelectasis, pleural effusion and chylothorax between the two groups, the number of children in the open group was greater than that in the thoracoscopy group, and the total postoperative complication rate (45.16%) was higher than that in the thoracoscopy group (36.17%).The recurrence rate and mortality of children in the thoracoscopy group were not significantly different from those in the open group (*P* > 0.05) (Table 3). In the open group, 62 children were followed up for 1 month to 9.7 years, with a median follow-up time of 4.31 years. Except for 7 deaths, 49 children recovered well without death or serious complications, and 6 children were lost to follow-up. In the thoracoscopy group, 47 children were followed up for 1 month to 9.5 years, with a median follow-up time of 5.24 years. 4 cases relapsed, 37 cases recovered well without death or serious complications, and 6 cases were lost to follow-up.

**Table 2:** Comparative analysis of operative-related indexes between the open group and thoracoscopy group (n = 109)

Operative related indexes	Open group (n = 62)	Thoracoscopy group (n = 47)	*P* value
Operation time (min)	103.66 ± 36.25	84.66 ± 36.35	0.000
Intraoperative blood loss (ml)	12.98 ± 9.47	7.71 ± 6.11	0.000
Total length of stay (days)	22.75 ± 11.84	17.95 ± 5.79	0.000
Intraoperative blood transfusion cases (cases)	4	5	0.432
Postoperative mechanical ventilation time (days)	9.57 ± 6.45	5.09 ± 2.74	0.000
Postoperative hospital stay (days)	21.39 ± 9.87	13.53 ± 4.23	0.000
Postoperative CCU admission time (days)	16.31 ± 9.92	6.21 ± 3.20	0.000

**Table 3:** Comparative analysis of postoperative complications between the open group and thoracoscopy group (n = 109)

Postoperative complications	Open group (n = 62)	Thoracoscopy group (n = 47)	*P* value
Incision infection (cases)	1	0	1.000
Incision dehiscence (cases)	1	0	1.000
lung infection (cases)	7	2	0.186
Respiratory failure (cases)	5	7	0.259
Pleural effusion (cases)	6	2	0.282
Atelectasis (cases)	4	1	0.285
Chylothorax (cases)	2	0	0.602
Pneumothorax (cases)	2	5	0.118
Recurrence (cases)	2	4	0.231
Mortality (cases)	7	0	0.017

## Discussion

CDH is a congenital disease that causes a defect in the diaphragm due to abnormal embryonic development, causing abdominal organs to herniate into the thoracic cavity and causing a series of pathophysiological changes. Generally, CDH occurs more often on the left side than the right side with the incidence ratio on the left and right sides being approximately 6:1, and CDH is rare on both sides with an incidence rate of approximately 2% [7]. In this study, the number of children with CDH on the left side (81.65%) was far greater than that of children with CDH on the right side (18.35%), which was consistent with literature reports. The incidence of CDH combined with other malformations is 30–70%, including cardiovascular formations (27.5%), genitourinary system malformations (17.7%), skeletal muscle system malformations (15.7%) and central nervous system malformations (9.8%) [8]. In this group, congenital pulmonary dysplasia (53, 48.62%) and congenital heart disease (45, 41.28%) were the main malformations in this study. Other combined malformations included cryptorchidism, pectus excavatum and right shifted heart, which was consistent with literature reports.

The traditional treatment for CDH is transthoracic or transabdominal diaphragmatic hernia repair. In the open group, 56 patients underwent transabdominal diaphragmatic hernia repair, and 6 patients underwent transthoracic diaphragmatic hernia repair. We believe that transabdominal surgery has the following advantages: easy to reset abdominal organs; easy to suture the edge of the diaphragm; and easy to deal with abdominal deformities, such as intestinal rotation malrotation. Therefore, transabdominal surgery is recommended for left-side diaphragmatic hernias. Transthoracic surgery is used for right-side diaphragmatic hernia due to liver obstruction because the diaphragm is clearly exposed during transthoracic surgery. Additionally, transthoracic surgery addresses thoracic adhesions and other combined chest deformities. However, in the open group, 5 children with right CDH without thoracic malformations were treated with transabdominal diaphragmatic hernia repair, which also achieved satisfactory clinical results. Therefore, we believe that the choice of approach is mainly based on the characteristics of the patient’s CDH lesions and which approach the surgeon is more familiar with. In this study, 47 patients underwent thoracoscopic diaphragmatic hernia repair. A purse-string suture with a needled suture was used to repair the diaphragmatic defect, close the diaphragmatic hernia, restabilize and suture the edge of the defect with barbed sutures. We believe that compared with ordinary absorbable sutures, continuous suturing of the diaphragm with barbed sutures has the following advantages: less bleeding, absorbability, no knot response, tight sutures and no knots during the suture process, which greatly shortens the operation time. Moreover, re-strengthening the diaphragm reduces recurrence.

With the development of MIS, thoracoscopy has gradually been used in the treatment of CDH. We compared the effects of open surgery and thoracoscopy in the treatment of CDH in children. The operation time, postoperative mechanical ventilation time, postoperative hospital stay and postoperative CCU admission time in the thoracoscopy group were shorter than those in the open group. Therefore, thoracoscopic treatment of CDH has the advantages of less bleeding and faster recovery than traditional open surgery. However, the results of this analysis may be affected by selection bias. In general, we give priority to thoracoscopic surgery, and if the operation is difficult, we will switch to open surgery. When the patient's condition is severe and the diaphragm defect is large, surgeons are more inclined to use open surgery to repair the defect.

This study compared the incidence of complications of thoracoscopy and open surgery. The literature reports that the most common complications of diaphragm hernia repair include intestinal obstruction, pneumothorax, pleural effusion and atelectasis. Other studies have reported scoliosis and pectus excavatum after open surgery [9]. In this study, the overall incidence of postoperative complications in the open group (45.16%) was higher than that in the thoracoscopy group (36.17%), which was equivalent to the incidence reported in the literature [10]. In this study, the main complications of the open group were lung infection, pleural effusion and respiratory failure. The main complications of the thoracoscopy group were respiratory failure and pneumothorax. The incidence of complications in the open group was higher than that in the thoracoscopy group, but there was no significant difference between the two groups (*P* > 0.05).There were 6 cases of pleural effusion and 7 cases of pneumonia in the open group. Open surgery has a larger traumatic area than thoracoscopic surgery and more exudation, which is likely to cause pleural effusion. Moreover, children are young and have poor resistance to lung infections. Two children in the open group died of respiratory failure after surgery. The possible causes were that the children had severe pulmonary dysplasia, poor cardiopulmonary function after surgery and could not tolerate open surgery [11].

This study compared the postoperative recurrence rate of the thoracoscopy and open groups. The recurrence rate of the thoracoscopy group (8.51%) was higher than that of the open group (3.23%), but the difference was not statistically significant. Some scholars have compared open surgery and thoracoscopic surgery, suggesting that the recurrence rate after thoracoscopic surgery for CDH is higher [12, 13] and that intraoperative hypercapnia and acidosis are more serious. The recurrence rate of thoracoscopic diaphragmatic hernia repair is reported to be between 0 and 25%. Most authors have reported that the recurrence rate is higher than 15% [14, 15]. The recurrence rate in this study was lower than that reported in the literature, which may be attributed to the thoracoscopic diaphragmatic hernia repair being only performed in patients with a small diaphragm defect (defect A/B/C). If a large defect was found under thoracoscopy, it was converted to open repair. In the open group, there were 2 cases of recurrence, which may have been due to poor development of the diaphragm, suture not reaching the edge of the normal diaphragm and a long-term increase in intra-abdominal pressure. The 2 cases of recurrence may have also been related to the large defect of the diaphragm, high tension of the diaphragm suture and loose suture. There were 4 cases of recurrence in the thoracoscopy group. We found that all recurrences occurred in the early stage of the use of thoracoscopy. The postoperative recurrence may have been related to several factors. (1) The surgical operation was performed under thoracoscopy, and the surgical field was enlarged. In addition, the surgeon could not accurately estimate the suture distance. As a result, the suture distance of each stitch was large, and the defect could not be closed well. (2) The diaphragm muscle tension was too large when sutured, and the sutured diaphragm muscle tissue was not thick enough, causing the diaphragm to tear. (3) The use of continuous sutures led to loose knots. In the later period, a purse-string suture with needled suture was used to repair the diaphragmatic defect, close the diaphragmatic hernia, restabilize and suture the edge of the defect with barbed sutures to achieve better clinical results. Scholars at home and abroad have also proposed the use of special equipment to assist in knotting and have achieved good results [16]. One of the major limitations of this study was the retrospective nature, which may be biased when collecting data. The second limitation in this study was that it was not a randomized study, and selection bias may have existed.

## Conclusions

Thoracoscopic surgery and open surgery can effectively treat CDH.Compared with conventional open surgery, thoracoscopy has the advantages of shorter operation time, less trauma, faster recovery and fewer complications.We believe that thoracoscopic surgery for type A/B diaphragmatic defect has certain advantages, but there is a risk of recurrence.

## Data Availability

The datasets used and analyzed during the current study are available from the corresponding author upon reasonable request.

## Abbreviations

CDH: Congenital diaphragmatic hernia
CCU: Coronary care unit
CT: Computed tomography
MIS: Minimally invasive surgery
